# Immunologic testing of xeno-derived osteochondral grafts using peripheral blood mononuclear cells from healthy human donors

**DOI:** 10.1186/1471-2474-6-36

**Published:** 2005-06-29

**Authors:** Vincent J Hetherington, Jill S Kawalec, Douglas S Dockery, Oleg S Targoni, Paul V Lehmann, Daniel Nadler

**Affiliations:** 1 Ohio College of Podiatric Medicine, 10515 Carnegie Avenue, Cleveland, Ohio, 44106 USA; 2 Cellular Technology Limited, 10515 Carnegie Avenue, Cleveland, Ohio, 44106 USA; 3 Centerpulse Orthopedics, Ltd., Postfach 65, CH-8404 Winterthur, Switzerland

## Abstract

**Background:**

One means of treating osteoarthritis is with autologous or allogeneic osteochondral grafts. The purpose of this study was to evaluate the innate immunological response in humans toward xeno-derived osteochondral grafts that have been partially or entirely treated by the photooxidation process.

**Methods:**

The antigens tested included bovine, porcine, ovine and equine osteochondral samples that have been treated in successive steps of photooxidation. ELISPOT assays were used to evaluate the production of IL-1, IL-4, IL-6, IL-10, IL-12 and TNF-α by human monocytes in response to the antigens.

**Results:**

Results indicated vigorous production of IL-1, IL-6, IL-10 and TNF-α in response to untreated bovine, porcine and equine specimens. This indicates that these samples are perceived as foreign, or stimulatory, by the human monocytes. There was no induction of IL-4 or IL-12, which is required for Th2 and Th1 immunity, respectively. In contrast, the processed bovine, porcine and equine samples did not induce significant activation of cells of the innate immune system. This occurred after the first step in processing (after cleaning in increasing strengths of ethanol). This suggests that the processing steps dramatically, if not completely, negated the immunostimulatory properties of the test sample. The results for the ovine samples indicate a reverse response.

**Conclusion:**

The findings of the study suggest that photooxidized bovine, porcine or equine samples have the potential to be used as an osteochondral graft. Although the first step in processing reduced the immunological response, photooxidation is still necessary to retain the structure and mechanical integrity of the cartilage, which would allow for immediate joint resurfacing.

## Background

There are various methods for repairing cartilage defects in patients with osteoarthritis or other degenerative joint disease. One such method is mosaicplasty, which consists of the use of osteochondral grafting with autogenous [1-3] or allogeneic [4-6] implants. Concerns exist, however, with the use of both autogenous grafts due to the need to harvest the osteochondral implants from a joint or part of a joint that is otherwise healthy, and for allogeneic materials with respect to disease transmission. Both of these graft types also have a limited availability.

There is the potential for using photooxidized xenogeneic osteochondral scaffolds in place of autogenous or allogeneic grafts. Photooxidation is a light-mediated process that results in the cross-linking of collagen fibers. The result is a material that is relatively non-immunogenic and resistant to chemical and enzymatic degradation, while maintaining the mechanical properties of the tissue, as demonstrated with bovine [7-10] and porcine [11,12] tissue. The photooxidized cartilage has been shown to have no viable chondrocytes after treatment. [10]

Animal studies have included analysis of photooxidized bovine pericardial tissue for replacement of the mitral valve [13] and aortic valve. [14] In addition, the benefits of photooxidation in vein grafting have been demonstrated. [15,16] Additional animal studies have demonstrated good biocompatibility with integration of xeno-derived osteochondral scaffolds. [10,17,18] Kawalec et al. [17] examined the use of photooxidized bovine osteochondral plugs in a rabbit model. The grafts were implanted into the patellar groove of both knees for 12 weeks. Histology indicated a chronic inflammatory reaction to the photooxidized implant. Active bone remodeling was observed in the bone portion of the implant, while the cartilage layer remained intact and undamaged, but covered by a thin layer of fibrous tissue. Akens et al. [18] implanted photooxidized xenogeneic osteochondral plugs into the femoral condyle of ovine knees and compared the findings to untreated xenogeneic and autogeneic grafts after six, 12 and 18 months. Macroscopically, results indicated that most grafts possessed a well-maintained cartilage surface at all time points. Histologically, sections from all three experimental groups showed evidence of cystic lesions at six months, in addition to evidence of bone remodeling. The number of sections containing cysts decreased at 12 months for the photooxidized samples, but remained high for the untreated xenogeneic and autogeneic samples. This was also observed at 18 months. Cartilage fusion between graft and host was observed for the photooxidized implants, but not for the untreated xenogeneic and autogeneic implants.

The purpose of this study was to evaluate the innate immunological response in humans toward xeno-derived osteochondral grafts that have been partially or entirely treated by the photooxidation process. The long-term goal of this research is to evaluate the potential for using photooxidized xeno-derived osteochondral grafts in the repair of articular cartilage defects.

## Methods

### Antigen preparation

The grafts tested in this project were made from bovine, porcine, ovine or equine bone and cartilage. Samples were prepared by removing an osteochondral plug approximately 8 mm in diameter by 7 mm in length. After retrieval, the samples were placed in a phosphate-buffered saline (PBS) solution to prevent them from drying out prior to processing. There were five groups of grafts, with the exception of the equine grafts, of which there were two groups, based on the specific steps performed during processing, as described in Table 1. Samples from each group were then frozen at -80°C, ground to a fine powder averaging 2–5 μm and subsequently lyophilized and packaged prior to testing. The antigens were dissolved in water at a concentration of 1 mg/ml and were used for the ELISPOT assays at 10 μg/ml.

**Table 1:** Experimental groups. Five groups of antigens, based on specific steps performed during processing

Processing steps	Bovine Group	Porcine Group	Ovine Group	Equine Group
Group 1 – untreated	B1	P1	O1	E1
Group 2 – after cleaning in increasing strengths of ethanol	B2	P2	O2	N/A
Group 3 – after cleaning in ethanol and methylene chloride	B3	P3	O3	N/A
Group 4 – after photooxidation process	B4	P4	O4	N/A
Group 5 – after sterilization with Gamma radiation	B5	P5	O5	E5

### Human PBMC donors

Peripheral blood mononuclear cells (PBMC) were obtained from ten healthy donors that were 30–50 years of age, and ten donors that were 50–70 years of age. There was an equal distribution of males and females. Heparinized blood was obtained by venipuncture. The study was performed under the approval of the Institutional Review Board at the Ohio College of Podiatric Medicine.

### Cell separations

The monocytes were isolated from PBMC by negative selection using the RosetteSep enrichment cocktail (Stemcell Technologies, Vancouver, BC, Canada). The enrichment cocktail was mixed with the blood to constitute a 50 μg/ml final concentration, followed by 20 minutes incubation at room temperature. Subsequently, the cells were separated using standard Ficoll-Hypaque density gradient centrifugation. The ensuing isolated monocyte enriched cell fraction was of >92% purity.

### ELISPOT assays

Human cytokine ELISPOT assays for the detection of Interleukin-1 (IL-1), IL-4, IL-6, IL-10, IL-12 and Tumor Necrosis Factor-alpha (TNF-α) were performed as described previously [19]. Briefly, ImmunoSpot plates (Cellular Technology Limited, Cleveland, OH) were coated overnight at 4°C with the cytokine-specific capture antibody in PBS at concentrations specified below. The plates were blocked with 1% bovine serum albumin (10 g/l in PBS: PBS-BSA) for one hour and washed three times with PBS. Monocytes were plated in triplicates in complete RPMI 1640 medium at 5 × 10^4 ^cells per well. Antigens were added at 10 μg/ml to the final concentration. Wells containing the cells in the complete medium were used as a negative control. For the bovine and porcine samples tested with blood from the 30–50 year old age group, lipopolysaccharide (LPS) at a concentration of 2 μg/ml was used as a positive control. For all other assays, phytohemaglutinin (PHA) stimulation at a final concentration of 10 μg/ml was used as a positive control.

The cells were cultured in an incubator at 37°C. The IL-1, IL-4, IL-6 and TNF-α assays cultured for 24 hours, while the IL-10 and IL-12 assays were harvested after 48 hours. Subsequently, the plates were washed three times with PBS, then three times with PBS-Tween (0.5 %), and the detection antibodies (in PBS-1% BSA-0.05%Tween) were added at concentrations specified below. After an overnight incubation at 4°C, plates were washed four times with PBS-Tween, and the Streptavidin-Horse Radish Peroxidase (HRP) conjugate (Dako Corp., Carpenteria, CA) was added at a 1:2000 dilution in PBS-BSA for 2 hours at room temperature. Afterwards, plates were washed three times with PBS-Tween followed by washing three times with PBS.

The spots were visualized using the HRP-substrate 3-amino-9-ethylcarbazole (AEC) (Pierce Pharmaceuticals, Rockford, IL). The AEC stock solution was prepared by dissolving 10 mg AEC in 1 ml N,N-dimethyl formamide (Fisher Scientific, Fair Lawn, NJ). For the actual development, 1 ml of the AEC stock solution was freshly diluted into 30 ml of 0.1 M sodium-acetate buffer (pH 5.0), filtered (0.45 μm), and mixed with 15 μl H_2_O_2_. Two hundred μl of the AEC solution were plated per well. The reaction was stopped by rinsing with distilled water when spots became clearly visible macroscopically (10 to 45 minutes, dependent on the cytokine). The plates were air-dried overnight before subjecting them to image analysis using a Series 2 ImmunoSpot Analyzer (Cellular Technology, Ltd., Cleveland, OH). Each measurement was made in triplicate, and averaged to obtain the final value.

For human ELISPOT assays, the anti- IL-4, IL-6, IL-10, IL-12 and TNF-α antibodies that were used were purchased from BD Pharmingen (San Diego, CA), while anti-IL-1 antibodies were received from Pierce Biotechnology (Rockford, IL). Capture antibodies were used as follows: anti-IL-1(cat. #M-421B-E) at 3 μg/ml, IL-4 (cat. #554515) at 5 μg/ml, IL-6 (cat. #554543) at 1.5 μg/ml, IL-10 (cat. #554705) at 5 μg/ml, IL-12 (cat. #555065) at 5 μg/ml, and TNF-α at 2 μg/ml (cat. #551220). For detection, biotinylated antibodies were used: IL-1 (cat. #M-420B-E) at 0.3 μg/ml, IL-4 (cat. #554483) at 1.5 μg/ml, IL-6 (cat. #554546) at 1 μg/ml, IL-10 (cat. #554499) at 1 μg/ml, IL-12 (cat. #554660) at 0.6μg/ml, and TNF-α at 1 μg/ml (cat. #554511).

## Results

### Bovine samples

**Figure 1:**
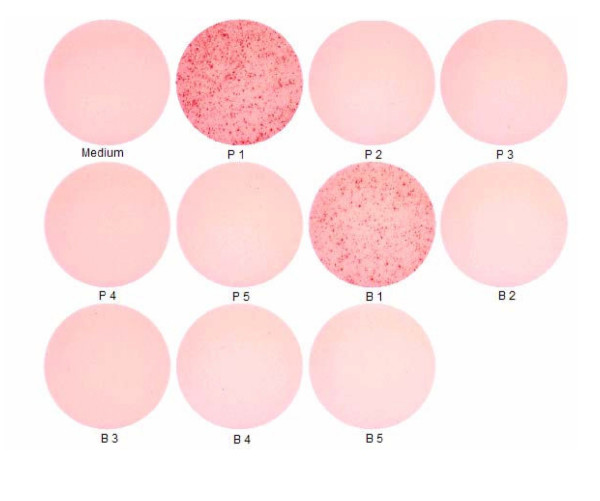
**ELISA spot wells. **Representative photograph of individual ELISA spot wells. Each black spot represents a cytokine-producing cell. This example represents IL-10 production by PBMC of healthy donors.

A representative photograph of individual ELISA spot wells is shown in Figure 1. Each black spot represents a cytokine-producing cell.

**Figure 2:**
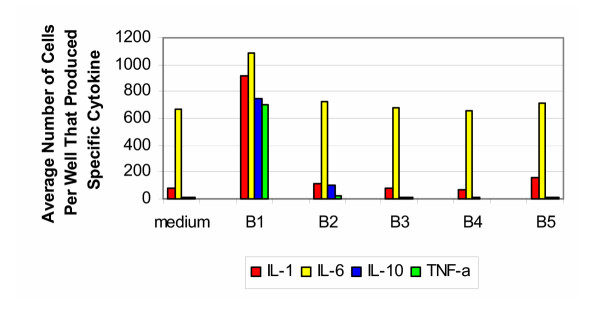
**Bovine 30–50 years old. **Production of cytokines by purified PBMC of healthy donors from 30–50 years old group (bovine).

The results of the ELISA spot testing for bovine samples tested with cells from donors 30–50 years of age are shown in Figure 2. Untreated samples of bovine bone and cartilage (group B1) induced vigorous production of IL-1, IL-6, IL-10 and TNF-α; however no induction of IL-4 or IL-12 was seen. In contrast, the processed bone and cartilage (groups B2–B5) did not induce significant activation of cells of the innate immune system. The elevated levels of IL-6 seen in all five groups were at a level similar to that found in the media alone. For the positive controls, stimulation with LPS resulted in a response on the order of that seen with group B1.

**Figure 3:**
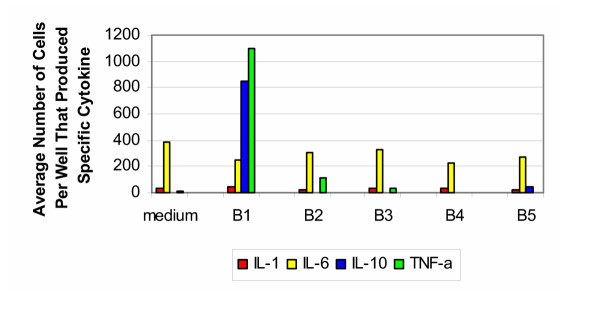
**Bovine 50–70 years old. **Production of cytokines by purified PBMC of healthy donors from 50–70 years old group (bovine).

The results of the ELISA spot testing for bovine samples tested with cells from donors 50–70 years of age are shown in Figure 3. The findings were very similar to that produced by the 30–50 year old group. The exception was the decreased production of IL-1 and IL-6 in response to untreated samples of bone and cartilage (group B1). For the positive control, stimulation with PHA resulted in cells producing IL-1 and IL-6 too numerous to count, while the number of cells producing IL-10 and TNF-α were on the order of that produced by group B1.

### Porcine samples

**Figure 4:**
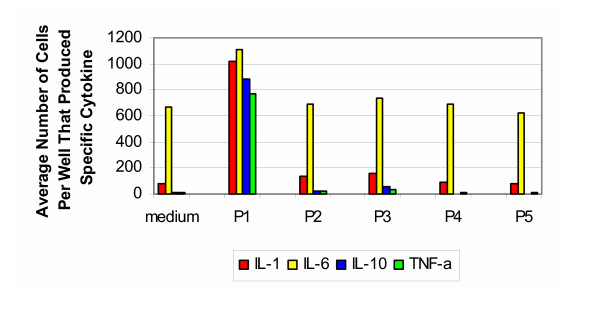
**Porcine 30–50 years old. **Production of cytokines by purified PBMC of healthy donors from 30–50 years old group (porcine).

The cytokine signature produced by testing porcine samples with cells from the 30–50 year old group (Figure 4) is similar to the findings from the bovine samples tested with cells from the same age group. In response to the untreated porcine bone and cartilage (group P1), there was a vigorous production of IL-1, IL-6, IL-10 and TNF-α, while no induction of IL-4 or IL-12 was seen. As with the bovine samples, the processed specimens (groups P2–P5) did not induce the significant response seen with the untreated samples. For the positive control, stimulation with LPS resulted in a response on the order of that seen with group P1.

**Figure 5:**
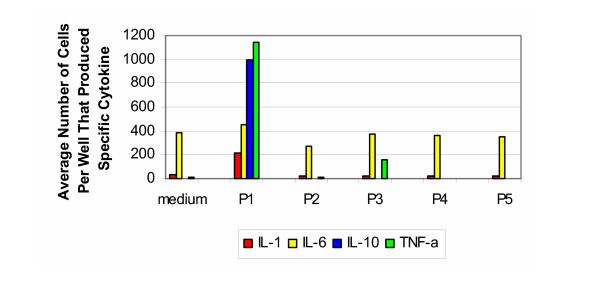
**Porcine 50–70 years old. **Production of cytokines by purified PBMC of healthy donors from 50–70 years old group (porcine).

The results for the porcine samples tested with cells from the individuals that were 50–70 years of age (Figure 5) were similar to those from the bovine samples tested with cells from the same group. A strong response was evoked towards the untreated specimens (group P1), with respect to IL-10 and TNF-α. While less than that produced by the younger age group, the production of IL-1 and IL-6 was still significant. As with the bovine results, the processed specimens (groups P2–P5) did not induce a vigorous response. For the positive controls, stimulation with PHA resulted in cells producing IL-1 and IL-6 too numerous to count, while the number of cells producing IL-10 and TNF-α were on the order of that produced by group P1.

### Ovine samples

**Figure 6:**
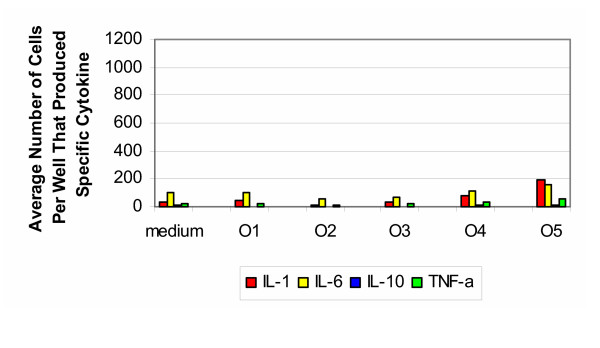
**Ovine 30–50 years old. **Production of cytokines by purified PBMC of healthy donors from 30–50 years old group (ovine).

The results for the ovine samples tested with cells from the 30–50 year old group (Figure 6) were the reverse of those from the bovine and porcine samples. Unprocessed samples (group O1) did not induce production of cytokines. Only after sterilization with Gamma radiation (Group O5) was there a slight increase in the production of IL-1 and IL-6. The production of IL-10 was much weaker than that resulting from bovine and porcine samples. For the positive controls, stimulation with PHA resulted in an average number of cells producing IL-1 and IL-6 too numerous to count, while the average number of cells producing IL-10 and TNF-α was also elevated in comparison to the negative control and experimental groups.

**Figure 7:**
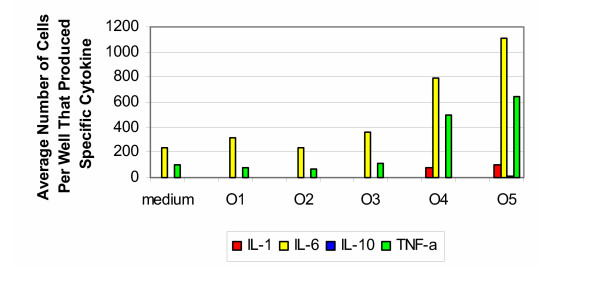
**Ovine 50–70 years old. **Production of cytokines by purified PBMC of healthy donors from 50–70 years old group (ovine).

The cytokine signature for ovine samples tested with cells from the 50–70 years of age group (Figure 7) was similar to that from the younger age group. There was no cytokine response to the untreated samples (group O1). After the photooxidized process (group O4), there was an increase in production of IL-1 and a vigorous production of IL-6 and TNF-α. After sterilization with Gamma radiation (group O5), there was an even more vigorous production of IL-6 and TNF-α. Again, the production of IL-10 was much weaker than that observed with bovine and porcine samples. For the positive controls, stimulation with PHA resulted in elevated responses for IL-1, IL-6, IL-10 and TNF-α, with respect to the negative control and experimental groups.

### Equine samples

**Figure 8:**
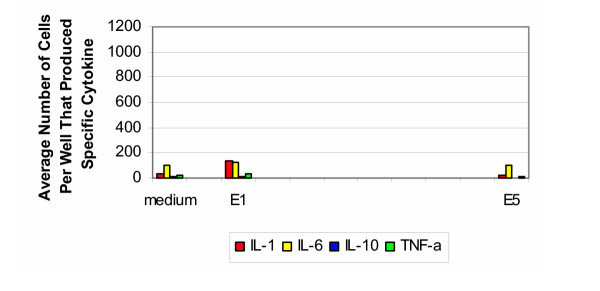
**Equine 30–50 years old. **Production of cytokines by purified PBMC of healthy donors from 30–50 years old group (equine).

Only two equine samples were tested: untreated specimens (group E1) and after Gamma radiation (group E5). The results for the equine samples tested with cells from the 30–50 year old age group are shown in Figure 8. In response to the untreated samples, there was a slight increase in the production of IL-1, IL-6 and TNF-α. This response was absent after sterilization with Gamma radiation. There was no production of IL-10 for either group. For the positive controls, stimulation with PHA resulted in cells producing IL-1 and IL-6 too numerous to count. The number of cells producing IL-10 was two orders of magnitude greater than group H1 and those producing TNF-α was one magnitude of order greater than that produced by group H1.

**Figure 9:**
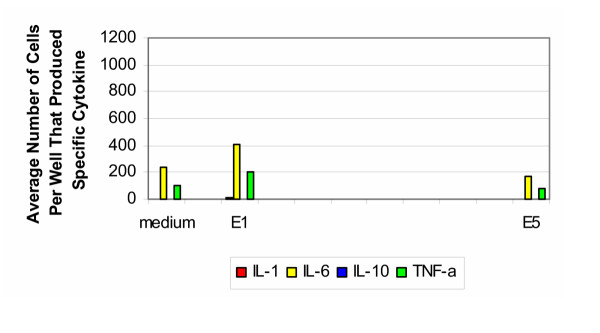
**Equine 50–70 years old. **Production of cytokines by purified PBMC of healthy donors from 50–70 years old group (equine).

The cytokine signature for equine samples tested with cells from the older age group (Figure 9) was similar to that from the younger age group. In response to untreated samples, there was production of IL-1, IL-6 and TNF-α. This response did not appear after sterilization with Gamma radiation. It should be noted that there was an absence in the production of IL-10 for both groups. For the positive controls, stimulation with PHA resulted in elevated responses for IL-1, IL-6, IL-10 and TNF-α, with respect to the negative control and experimental groups.

## Discussion

The cytokine signature produced by the untreated bovine and porcine samples is consistent with vigorous activation of cells of the innate immune system. This pertains primarily to cytokines involved in induction of a humoral immune response (IL-10), but not of cytokines that are required for Th1 immunity (IL-12) or Th2 immunity (IL-4). The IL-1 and TNF-α production seem to favor mixed Th0-type T cell responses. This indicates that the untreated bovine and porcine samples are perceived as foreign, or stimulatory, by the human cells. This is consistent in both age groups, and most individuals tested behaved comparably. Therefore, in spite of genetic differences in the human test population, rather uniform response patterns were seen, suggesting that the findings will broadly apply to the human population.

In contrast, the processed bovine and porcine osteochondral samples did not induce the production of cytokines. This was observed after the first processing step, after cleaning in increasing strengths of ethanol. This suggests a highly improved biocompatibility, with respect to inducing a rejection T cell/antibody response and inducing sterile granulomatous rejections in the sense of a foreign body reaction. It can be expected that these non-stimulatory samples can be considered well suited for transplantation. Again, this was observed for both age groups.

The cytokine signature for equine samples tested with cells from both age groups is similar to that observed with bovine and porcine samples. The exception with the untreated samples is the weak production of IL-10, which is a potent B cell differentiation factor. Thus, the equine samples may be less likely to induce antibody responses.

The reverse was seen in response to ovine samples. The untreated samples did not produce a response, while the specimens that were photooxidized, and to a greater extent those that were sterilized with Gamma irradiation resulted in a response of IL-1, IL-6 and TNF-α. This suggests a mixed Th0-type T cell response. There was a much weaker production of IL-10, which indicates the absence of a humoral response. These findings were observed with both age groups.

It should be noted that the graft material tested in this study was a prepared material that was subjected to a series of proprietary steps of cleaning, photooxidation and sterilization in order to develop a graft or scaffold that avoids human antigenicity and rejection reactions. The antigenic composition of the processed material (groups 2–5) is fundamentally different from the natural, unprocessed material (group 1). It was intended to test each graft as a whole, as it is proposed to be used in clinical applications, rather than breaking down the graft into its components. The grafts that have been photooxidized are not live material, and have been rendered hypo-immunogenic to an extent that they are accepted in a series of recipient animal species without the need for immune suppression. [10,17,18] Photooxidized materials have been used clinically in cardiac surgery, and, to the best of our knowledge, with the graft material tested as a whole and not its potential constituents. [11-16]

Previous work has established that photooxidized cartilage is accepted by the host better than untreated cartilage. [17,18] It is likely that the oxidation process chemically modifies the protein components in such a way that these structures stimulate cells of the innate immune system to induce, for example, prostaglandins. Frequently, sterile inflammation of this sort enhances wound repair and therefore enhances the vascularization and integration of the grafted tissue, rather than rejection. Because the clinical acceptance of these grafts is common we assume that such reactions are also induced in this model.

Monocytes are not constitutive parts of the joint, however these cells are rapidly recruited to the joint after tissue injury, including surgical implantation of grafts. Unlike chondrocytes that are not professional antigen presenting cells, monocytes can phagocytose, process and present graft antigens and initiate an immune response. This initial phase was evaluated in this study. Once the T cells are primed, they can interact with chondrocytes and other non-professional antigen presenting cells in the joint to exert the effector arm of the rejection reaction.

The conclusions reached in this study were drawn using in vitro experiments. The isolation of monocytes and their tissue culture plates might affect the functionality of the cells and modify them relative to their in vivo activation state. These constraints apply to any in vitro work. However, the purpose of this study was to establish whether monitoring the activity of such cells in vitro would provide insights on additional activation of them by stimulation with compounds such as the tissue culture material that was used. It is clearly shown that beyond whatever activation occurs in tissue culture, the graft materials tested induce a variable level of activation. Because activation of such cells of the innate immune system is key to their immunological activity, this study shows that sensitive monitoring of monocytes activity in response to such materials can be done in vitro.

## Conclusion

Grafts are rejected by a series of immune recognition events that include cells of the innate immune system (dendritic cells, macrophages) as well as the downstream activation of lymphocytes. Activation of cells of the innate immune system is a prerequisite for inducing an immune response by lymphocytes. Moreover, the type of cytokines induced in cells of the innate immune system by the antigen will define the (Th1/Th2) type of T cell response that evolves. This study demonstrated a reduction in the innate immune response to processed bovine, porcine and equine bone and cartilage, indicating a potential for use as a xeno-derived osteochondral scaffold. The effect was observed to occur after the first step in processing (after cleaning with increasing strengths of alcohol).

Although the first step in processing reduced the immunological response, photooxidation is still necessary to retain the structure and mechanical integrity of the cartilage, which would then allow for immediate joint resurfacing. The next phase of the study is to evaluate the downstream activation of the immune system caused by xeno-derived osteochondral grafts in an active system model, using an animal model. This will be followed by evaluation of the implant and immune reactions when a photooxidized implant is placed in the joint environment. Subsequent evaluation for the detection of viral or other residual infectious materials, such as α-galactase, is needed to alleviate concerns about xenotransplantation.

## Competing interests

The research project was funded by Centerpulse Orthopedics Ltd. It is not known whether they will gain financially from the publication of this paper.

## Authors' contributions

VJH helped conceive the study, participated in its design and coordination, assisted with analysis of findings, and helped to draft the manuscript. JSK participated in the design of the study, coordinated the blood withdrawal, assisted with data analysis, and drafted the manuscript. DD carried out blood withdrawal and assisted with analysis of the findings. OST coordinated the cell separations and ELISPOT assays, assisted with data analysis and helped to draft the manuscript. PVL participated in the design of the study, coordinated cell separations and ELISPOT assays and assisted with data analysis. DN carried out the antigen preparation. All authors read and approved the final manuscript.

## Pre-publication history

The pre-publication history for this paper can be accessed here:


